# Biomarkers associated with the diagnosis and prognosis of *Mycoplasma pneumoniae* pneumonia in children: a review

**DOI:** 10.3389/fcimb.2025.1552144

**Published:** 2025-03-18

**Authors:** Lele Ding, Yonghong Jiang

**Affiliations:** ^1^ Pediatrics, Longhua Hospital Affiliated to Shanghai University of Traditional Chinese Medicine, Shanghai, China; ^2^ Longhua Clinical Medical College, Shanghai University of Traditional Chinese Medicine, Shanghai, China

**Keywords:** biomarker, predictive model, *Mycoplasma pneumoniae*, child, diagnosis, prognosis

## Abstract

Community-acquired pneumonia (CAP) is a major cause of death in children, and *Mycoplasma pneumoniae* (MP) is the main pathogen of CAP in children in China. Although *Mycoplasma pneumoniae* pneumonia (MPP) is usually a self-limiting disease, many children develop multiple complications due to drug resistance or untimely diagnosis and treatment, and may even progress to severe MPP or refractory MPP with a poor prognosis. It is important to explore the value of biomarkers that can be used in clinical practice to assess the severity of pneumonia and assist in clinical decision making. In this article, we searched the literature in the last four years to review the roles of various types of biomarkers in MPP and the associated clinical predictive models, with the aim of helping pediatricians to understand the evaluation indexes related to MPP in children other than microbiology.

## 1 Introduction

Pneumonia is a serious global child health problem and the leading cause of death in children. *Mycoplasma pneumoniae* (MP) is the main pathogen causing community-acquired pneumonia in children aged 5 years and over (National Health Commission of the People’s Republic of China, 2023). According to statistics, the incidence of community-acquired pneumonia in children caused by MP infection in China is 15%-37%, with the highest incidence in the 5-10 age group (Tsai et al., 2021). Especially in the two years following the COVID-19 outbreak, the incidence of MPP has shown a clear upward trend in many countries (Dumke, 2024; Dungu et al., 2024). *Mycoplasma pneumoniae* pneumonia (MPP) possesses a degree of self-limiting characteristics, and the prognosis for treatment is typically favorable. However, some children experience a poor response to standard treatment and develop into refractory MPP (RMPP). Particularly following the COVID-19 pandemic, macrolide antibiotic-resistant MP infections have become more prevalent, resulting in an annual increase in the incidence of RMPP. Such children are prone to a combination of multiple intrapulmonary or extrapulmonary conditions, which may even lead to death in severe cases (Xu et al., 2024). Therefore, the early diagnosis of MPP and the accurate understanding of the condition are particularly important. The initial clinical symptoms in children with MPP are nonspecific. MP culture is the gold standard for diagnosis of the disease, with demanding and time-consuming conditions, while MP nucleic acid and antibody tests are more commonly used in clinical practice. However, there is a lack of sensitive markers for assessing the severity and prognosis of the disease. As our understanding of MPP has deepened in recent years, many biomarkers have been considered that are associated with the early recognition, determination of severity, and prognostic assessment of MPP. Moreover, with the advancement of Internet-based information technology, a range of predictive models has emerged. The application of these biomarkers can assist in optimizing clinical treatment decisions and reducing the incidence of poor prognoses. In this paper, we conducted a search in both English databases (such as PubMed) and Chinese databases (such as CNKI) using the keywords (“*Mycoplasma pneumoniae*” or “*Mycoplasma pneumoniae* infection” or “*Mycoplasma pneumoniae* pneumonia”) and (“children” or “adolescents” or “pediatrics”) and (“diagnosis” or “prognosis” or “severity” or “differentiation” or “value” or “biomarker”). We focused on biomarker literature related to MPP published between January 2021 and December 2024, carefully screening and organizing the results. Some important related articles published before the screening time limit were also included. This article mainly discusses 6 aspects: cell-based markers, protein-based markers, cytokine-based markers, nucleic acid-based markers, imaging-based markers, and multi-indicator joint predictive models.

## 2 Cell-based markers

### 2.1 Lymphocyte-associated ratio

In MPP children without bacterial infection, white blood cell (WBC) levels usually do not change significantly, mostly manifested as an increase in the percentage and count of neutrophils and monocytes, and a decrease in the percentage and count of lymphocytes (Zheng and Zhuo, 2021). Platelet is also involved in the immune response of the body after MP infection, resulting in increase in quantity and volume. In recent years, neutrophil-lymphocyte ratio (NLR), platelet-lymphocyte ratio (PLR), monocyte-lymphocyte ratio (MLR), mean platelet volume-to-lymphocyte ratio (MPVLR) have been recognized as important markers to reflect the immune-inflammatory status of the body. They are related to the prognosis of various types of tumors, cardiovascular and cerebral vascular diseases, and diabetes mellitus (Yang et al., 2021; Zhang et al., 2021; Sun and Li, 2022; Li et al., 2024b). In children with MPP, the levels of NLR, PLR, MLR, and MPVLR were significantly elevated, which were positively correlated with the severity of the disease and the pathological changes of lungs (He et al., 2024; Pei and Luo, 2024; Qiu, 2024; Wang and Shen, 2024; Wang et al., 2024). The high levels of NLR, as well as PLR, MLR, and MPVLR, suggested strong inflammatory reactions and platelet activation. In clinical practice, these indicators can be combined and observed to assist in the diagnosis of MPP and to determine the severity of the disease. In the identification of pathogens, Chen et al (Chen et al., 2024). compared a series of peripheral blood parameters between children with MPP and influenza. They found that PLR levels were significantly higher in children with influenza than in children with MPP. This suggests that PLR may have certain value in differentiating MPP from influenza. In addition, Chu et al (Chu et al., 2024). found that lymphocyte percentage (Lym%) (cut-off value: 22.1%) and neutrophil percentage (Neu%) (cut-off value: 65.2%) were effective in differentiating MPP and influenza A infections, and platelet distribution width (PDW) was effective in differentiating MPP and severe acute respiratory syndrome coronavirus 2 (SARS-CoV-2) infections, with a threshold of 15%. In terms of the prognosis of MPP, NLR, PLR and MPVLR are independent predictors of poor prognosis such as severe MPP (SMPP) and RMPP (Li et al., 2023; Jiang and Liu, 2024; Mu et al., 2024; Wang and Shen, 2024). For children with MPP over 6 years old, children with NLR >3.92 or MPVLR >5.29 are more likely to progress to RMPP, and the accuracy of prediction is higher than that of C reactive protein (CRP) (Ling et al., 2021).

In conclusion, as a basic test program, blood routine related indexes provide a new basis for early differential diagnosis of MPP and identification of children with high-risk prognosis. However, the use of test results at what point in the course of the disease as a reference and the associated thresholds need to be further investigated to determine. In particular, it is not known whether infants and preschool children, whose Neu% and Lym% vary from time to time, have different thresholds than older children.

### 2.2 Lymphocyte subset

**Figure 1:**
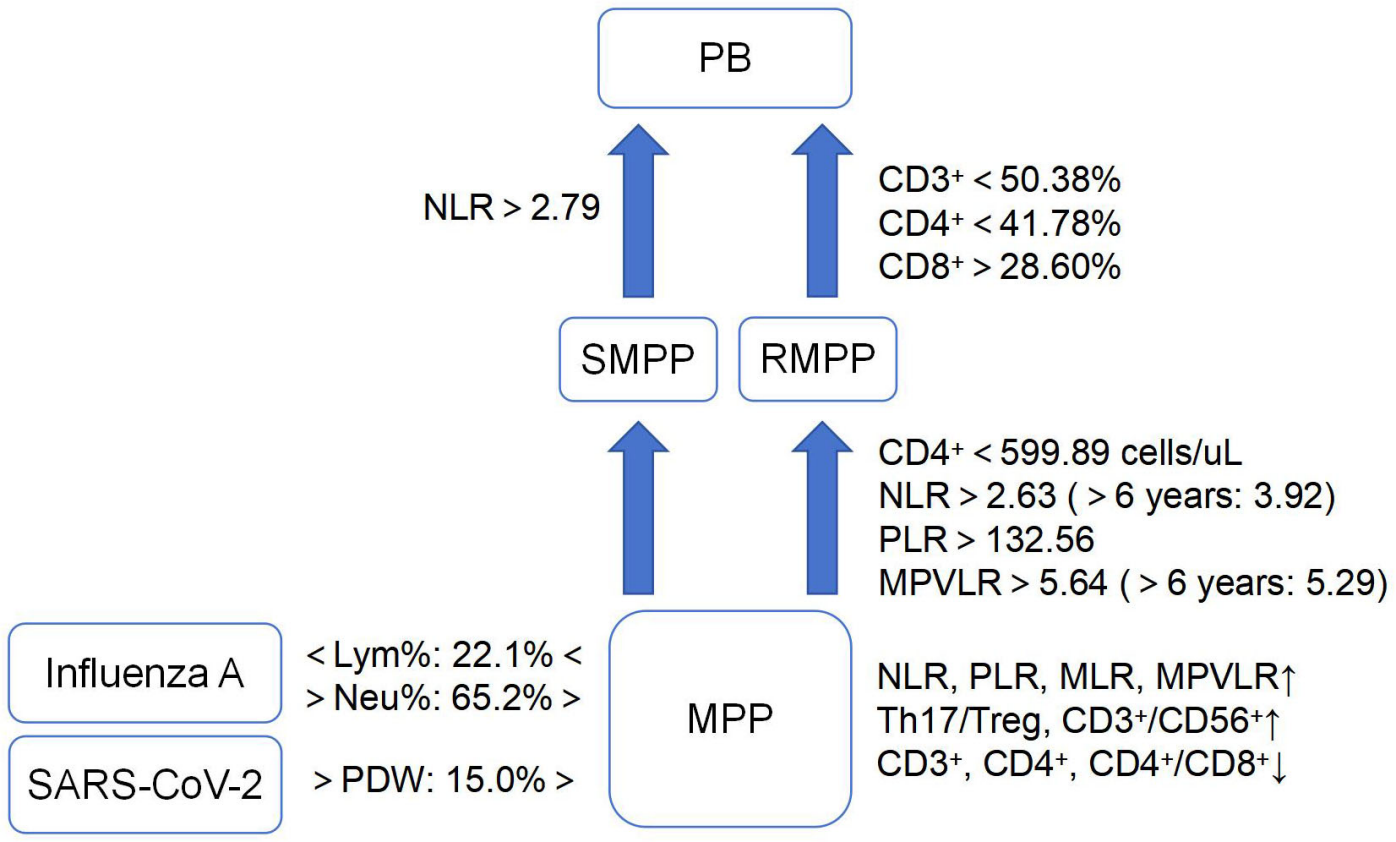
Characteristics of cell-based markers in MPP and its different prognoses.(MPP, *Mycoplasma pneumoniae* pneumonia; SMPP, severe MPP; RMPP, refractory MPP; PB, plasticoid bronchiolitis; SARS-CoV-2, severe acute respiratory syndrome coronavirus 2; NLR, neutrophil-lymphocyte ratio; PLR, platelet-lymphocyte ratio; MLR, monocyte-lymphocyte ratio; MPVLR, mean platelet volume-to-lymphocyte ratio; Lym%, lymphocyte percentage; Neu%, neutrophil percentage; PDW, platelet distribution width; Th17/Treg, T helper cell 17/regulatory T cell). ↑, increase; ↓, decrease.

Lymphocyte subsets are mainly related to the immune response of the body after MP infection, which is mainly characterized by a decrease in the levels of CD3^+^ and CD4^+^ as well as an increase in the levels of CD8^+^ and CD19^+^. Moreover, the levels of CD3^+^, CD4^+^, and CD4+/CD8+ were shown to be lower in the acute phase than in the recovery phase, and lower in severe than in mild of MPP (Jin et al., 2021; Jiang et al., 2022; Kong, 2022; Song, 2022; Wang and Gong, 2024). For early identification of RMPP, the predictive value of CD4^+^ count was superior to CD3^+^, CD19^+^, CD56^+^ and CD4^+^/CD8^+^, but the specific diagnostic cut-off value varied in different studies. In the study of Li et al (Li et al., 2019), CD4^+^ < 599.89 cells/µL has a high predictive value for RMPP, with sensitivity as high as 90% and an areas under curve (AUC) of 0.900 [95% confidence interval (95%CI): 0.852-0.948]. The cut-off value of CD4^+^ obtained by Yao et al (Yao et al., 2023). was relatively high, which was 1370 cells/µL, and the sensitivity was 85.94%. The specific and reliable cut-off value needs to be further studied and confirmed. Among T lymphocytes, both T helper cell 17 (Th17) and regulatory T cells (Treg) are differentiated from CD4^+^ T lymphocytes, the former mainly exerts pro-inflammatory effects through the secretion of IL-17, while the latter mainly exerts immunosuppressive effects to reduce the body’s immunity to MP through IL-10. With the progression of MPP, Th17/Treg, CD3^+^CD56^+^ showed a significant increase (Cao et al., 2022). In addition, children with MPP usually have varying degrees of decreased lung function. In infants and young children, there is a risk of developing asthma if not treated timely (Wang, 2023; Ha et al., 2024). It was shown that the levels of forced expiratory volume in one second (FEV1) and forced expiratory volume in one second/forced vital capacity (FEV1/FVC) in children with MPP were positively correlated with CD3^+^, CD4^+^, CD4^+^/CD8^+^ and negatively correlated with CD3^-^CD19^+^, CD19^+^CD23^+^. When CD3^+^ <50.38%, CD4^+^ <41.78%, or CD8^+^ >28.60%, children with RMPP had a higher probability of developing plasticoid bronchiolitis (PB), and the sensitivity of combining these three indicators to diagnose PB in children with RMPP was 87.50% (Wang and Wang, 2022). It can be seen that lymphocyte subsets are closely related to the onset and progression of MPP, and the severity of MPP children can be effectively evaluated by monitoring specific lymphocyte levels (**Figure 1**).

## 3 Protein-based markers

### 3.1 Acute phase protein

CRP, serum ferritin (SF), and serum amylase A (SAA) are all acute phase proteins originating from the liver. When the body is stimulated by MP infection, activated immune cells (such as macrophage and monocyte) release interleukin-6 (IL-6), interleukin-1β (IL-1β), and tumor necrosis factor-α (TNF-α), and these inflammatory factors reach the liver through blood circulation. IL-6 can bind to hepatocyte membrane receptors, activate the signal transducer and activator of transcription 3 pathway, and promote the expression of CRP and SAA genes. IL-1β and TNF-α can activate the nuclear factor kappa-light-chain-enhancer of activated B cells pathway to promote the expression of SAA gene. In addition, IL-6 and IL-1β can not only directly induce ferritin synthesis in hepatocytes, but also elevate intracellular ferritin levels by regulating iron metabolism (Mosquera-Sulbaran et al., 2021; Gehrer et al., 2023; Chang et al., 2025).

Among the three, SAA rises the most earliest, usually within 3-6 h, and the magnitude of the rise is larger than that of CRP. And it can be rapidly reduced to normal levels after the antigen was cleared. This makes it a sensitive indicator to reflect the infection and recovery of the organism (Sack, 2020). In children with pneumonia due to infection by different pathogens, the elevation of SAA levels in those with MP infection falls between that of viral and bacterial infections; furthermore, using 247.56 mg/L as the diagnostic threshold for MPP yields a sensitivity of up to 90.0% (Pan et al., 2024). In children with MP infection, changes in SAA levels positively correlate with CD8^+^ and negatively correlate with CD4^+^. SAA >203.56 mg/L had a sensitivity of 86.7% and a specificity of 83.3% for diagnosing MP infection, with an AUC of up to 0.924 (Jiang et al., 2022).

CRP generally begins to increase 6-8 h after infection, peaks within 48 h, and lasts longer than SAA during the inflammatory process (Sproston and Ashworth, 2018). “The guideline for the diagnosis and treatment of MPP in children (2023)” pointed out that CRP began to increase significantly after 3 days of fever in SMPP, and the degree of increase was positively correlated with the severity of the disease (National Health Commission of the People’s Republic of China, 2023). It was found that the level of CRP was lower in the MP group than in the bacterial group (Zhu and Guo, 2024). CRP =16.91 mg/L can be used as a cut-off value to differentiate MPP from bacterial pneumonia with a sensitivity of 75.8% and a specificity of 85.7% (Luo and He, 2024). However, it is difficult to perform a definitive identification of pathogens by applying CRP alone. The time of CRP elevation maybe advance and the magnitude of the elevation increases when MPP is combined with bacterial infection (Tan et al., 2024). Chen et al (Chen et al., 2024; Wang et al., 2024). found that CRP was a biomarker for predicting RMPP and SMPP, with cut-off values of 39.34 mg/L and 33.56 mg/L, respectively. Wang et al (Wang et al., 2024). divided the children with SMPP into two groups according to whether lung tissue necrosis occurred. They found that CRP >67.5 mg/L on days 6-10 of the disease course could identify the subtype of pulmonary necrosis early, with a sensitivity of 96% and a specificity of 89%. A study investigated the regression time of imaging lesions of 399 children with lobar pneumonia caused by MP infection, and found that the regression time of children with CRP ≥25.92 mg/L often exceeded 2 months (Zheng et al., 2024). In addition, CRP ≥12.27 mg/L and 76.73 mg/L were also independent risk factors for mucus plug formation in children with MPP and for pulmonary thrombosis in children with SMPP, respectively (Zhang et al., 2021; Yu et al., 2024). Meanwhile, CRP ≥137 mg/L can be used to predict the occurrence of PB in children with RMPP (Liu et al., 2024). In summary, CRP has limited value in identifying MP from pathogens such as bacteria and viruses, but has good value in assessing and predicting the severity of MPP.

**Figure 2:**
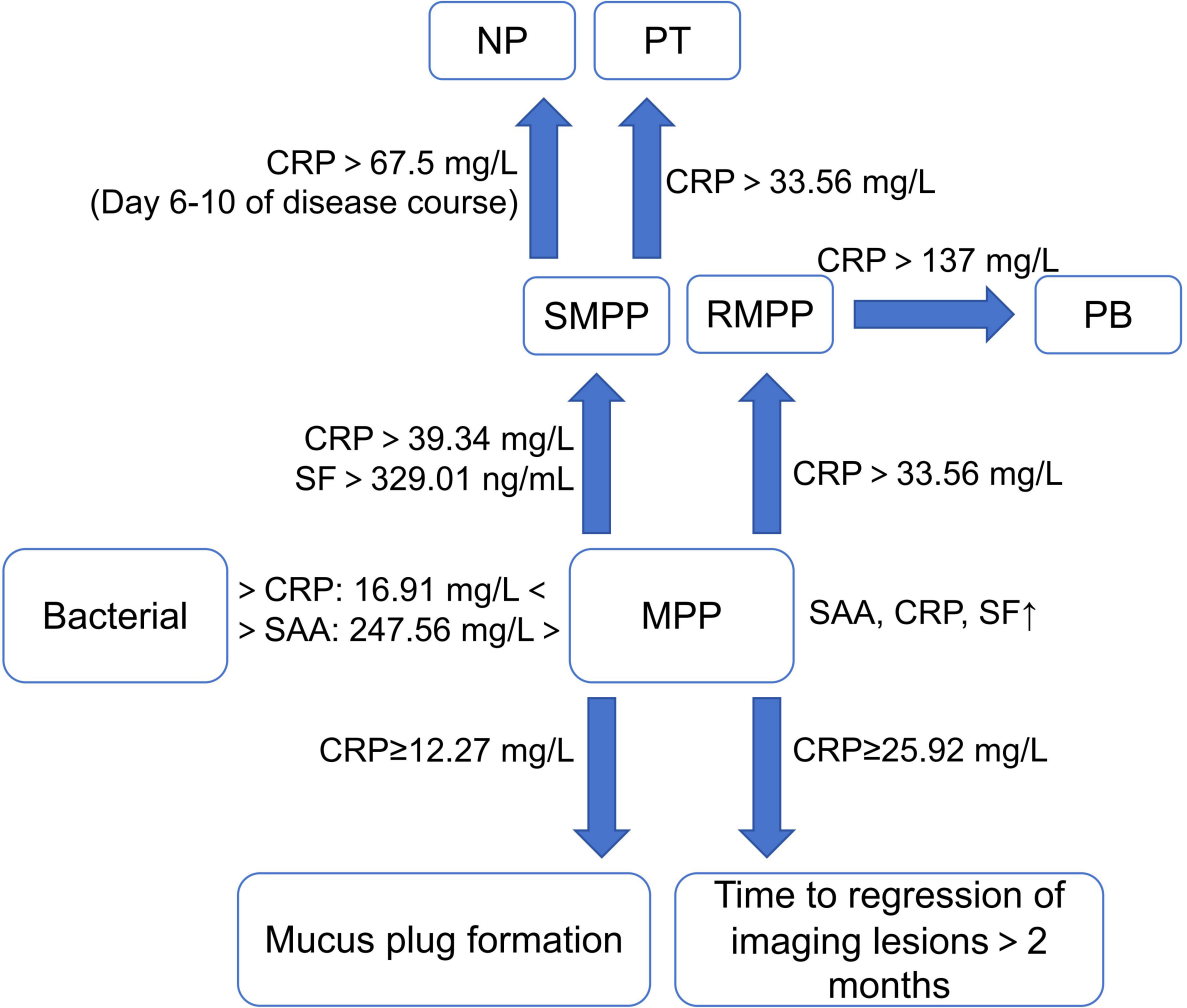
Characteristics of acute phase protein in MPP and its different prognoses. (MPP, Mycoplasma pneumoniae pneumonia; SMPP, severe MPP; RMPP, refractory MPP; NP, necrotizing pneumonia; PT, pulmonary thrombotic; PB, plasticoid bronchiolitis; SAA, serum amylase A; CRP, C reactive protein; SF, serum ferritin). ↑, increase.

Compared to CRP and SAA, SF has a delayed response, generally rising 24-48 h after infection and lasting for a long time. Wei et al (Wei et al., 2024). showed that SF was positively correlated with the severity of MPP. The increase in SF of children with RMPP is greater than that of children with general MPP (GMPP), and it can reflect the degree of pulmonary inflammation and tissue damage (Fu et al., 2022). Wen et al (Wen et al., 2021). showed that SF >329.01 ng/mL predicted RMPP with a specificity of 93.13% and an AUC of 0.90 (**Figure 2**).

### 3.2 Lactic dehydrogenase

LDH is a glycolytic enzyme that exists in almost all major organs of the body. When inflammatory damage occurs in lung tissue due to MP infection, LDH enters the blood with cell division or cell damage, resulting in an increase in the level of LDH (Yan et al., 2021). The magnitude of the increase in LDH is positively correlated with the severity of MPP (Wang et al., 2023a). LDH >354 U/L can predict the occurrence of SMPP in children (Qiu et al., 2022). LDH >379 U/L as the diagnostic cut-off value for RMPP has a sensitivity of 66.67% and a specificity of 93.91% (Chen et al., 2024). Wen et al (Wen et al., 2021). also achieved similar results. The regression of lung lesions in such children often took longer, which may take more than 8 weeks (Zheng et al., 2024). For children with macrolide-resistant mutations in macrolide-unresponsive MPP (MUMPP), LDH ≥399 U/L suggested that the risk of the children progressing to RMPP will increase significantly (Cheng et al., 2024). In terms of complications in MPP, LDH of 393.0 U/L can predict the occurrence of necrotizing pneumonia (NP) in children with MPP, with sensitivity and specificity of 68.3% and 86.2%, respectively. And as the LDH continued to rise, the risk of NP in children with MPP will continue to increase (Ren et al., 2024). LDH >462.65 U/L (Zhang et al., 2021) and >482 U/L (Zhong et al., 2021) were independent risk factors for mucus plug formation and PB in children with MPP, respectively. In addition, a high level of LDH is also an independent risk factor for SMPP combined with bronchiolitis obliterans (BO) (Zheng et al., 2022). In terms of treatment, LDH is significantly increased in children with RMPP requiring steroid hormone therapy, and the LDH level at the time of the child’s visit can be used to preliminarily determine whether the child with MPP needs to apply steroid hormones and the optimal dose (Wei et al., 2024). At the same time, when the child’s LDH reaches 1,114 U/L, it also prompts that the child may require oxygen inhalation at present, and the child’s vital signs need to be evaluated in time (Lee and Choi, 2022). It can be seen that similar to CRP, LDH has good value and high sensitivity in predicting severe cases and serious complications of MPP as well as guiding clinical treatment.

### 3.3 D-dimer

As a specific degradation product of fibrin, D-dimer mainly reflects the function of fibrinolytic system. When MP infects the organism, local tissue ischemia and hypoxia cause damage to vascular endothelial cell and collagen exposure, which will also activate the human fibrinolytic system and complement system, resulting in an increase in D-dimer. The clinical symptoms of MPP in children with high D-dimer levels are more serious and require a longer treatment cycle, which may be closely related to the severity of lung inflammation after MP infection (Zheng et al., 2021). Qiu et al. (2022) showed that D-dimer was a better predictor of SMPP than CRP and LDH in a study of 786 children with MPP. Children with D-dimer >0.31 mg/L (although it may be within the normal range) are more likely to have pleural effusion. And when D-dimer >0.40 mg/L, there is a high risk of SMPP. In addition, D-dimer >2.10 mg/L also has a clear diagnostic value for RMPP, with a specificity of 81.86% (Wen et al., 2021). Another study also found that D-dimer >7.33 mg/L was significantly related to MP-positive in pleural effusion. The clinical symptoms of such children are more serious than those with MP-negative in pleural effusion (Li et al., 2024).

In terms of complications in MPP, children with SMPP and RMPP should be alert to thrombosis if D-dimer is significantly increased (Fu et al., 2023). The further study showed that D-dimer >3.98 mg/L can be used as an independent predictor for pulmonary thrombotic (PT) in children with SMPP, with sensitivity and specificity higher than 90% and an AUC of 0.95 (Yu et al., 2024). “The guideline for the diagnosis and treatment of MPP in children (2023)” also indicated that chest pain and/or hemoptysis in children with MPP accompanied by D-dimer ≥5 mg/L will help to diagnose PT (National Health Commission of the People’s Republic of China, 2023). In addition, the significantly increased D-dimer correlates with NP in children with SMPP. D-dimer >3.705 mg/L can independently predict MPP combined with NP, with an AUC of 0.865. And the time to regression of imaging lesions in such children may be >3 months (Li et al., 2023; Luo et al., 2023). In conclusion, it can be seen that in children with MPP, D-dimer is mainly used for the prediction of PE and the assessment of the severity of MPP.

### 3.4 Procalcitonin

PCT is a calcitonin peptide substance secreted by thyroid C cells, consisting of 116 amino acid factors, and is a common systemic inflammatory biomarker. Severe pneumonia caused by bacteria usually has a significant increase in WBC, CRP and PCT in the early stage of the disease (Yin and Mo, 2022). Thus PCT serves as a specific biomarker of bacterial infection, whereas it is commonly used to identify bacterial and nonbacterial infections. Ruan et al (Ruan et al., 2024). compared the clinical characteristics of 506 children with MPP and 311 children with *Streptococcus pneumoniae* pneumonia (SPP), both MPP and SPP children had an increase in PCT. And the increase in children with SPP was greater. The reason may be that MP has lipopolysaccharide and endotoxin effects similar to Gram-negative bacteria, which induce macrophages and monocytes to secrete PCT. Some scholars also compared the level of PCT in children with pneumonia caused by bacteria, MP, and virus, and found that the magnitude of the elevation in the three groups was bacterial > MP > virus. PCT >0.605 ng/mL can effectively diagnose MPP (Pan et al., 2024). Among children with MPP, Jiang et al (Jiang et al., 2022). found that the level of PCT in the acute phase was higher than that in the recovery phase and in healthy children. And the sensitivity and specificity of 1.12 ng/mL as the diagnostic cut-off value of MP infection were higher than 70%. Meanwhile, PCT is also positively correlated with the severity of MPP, and severe cases than mild cases (Weng et al., 2022; Wang et al., 2024). As a common inflammatory marker, it can be seen that the main value of PCT in MPP is the early differential diagnosis of MPP, but the diagnostic thresholds of identifying pathogens need to be determined by further studies with large sample sizes.

**Figure 3:**
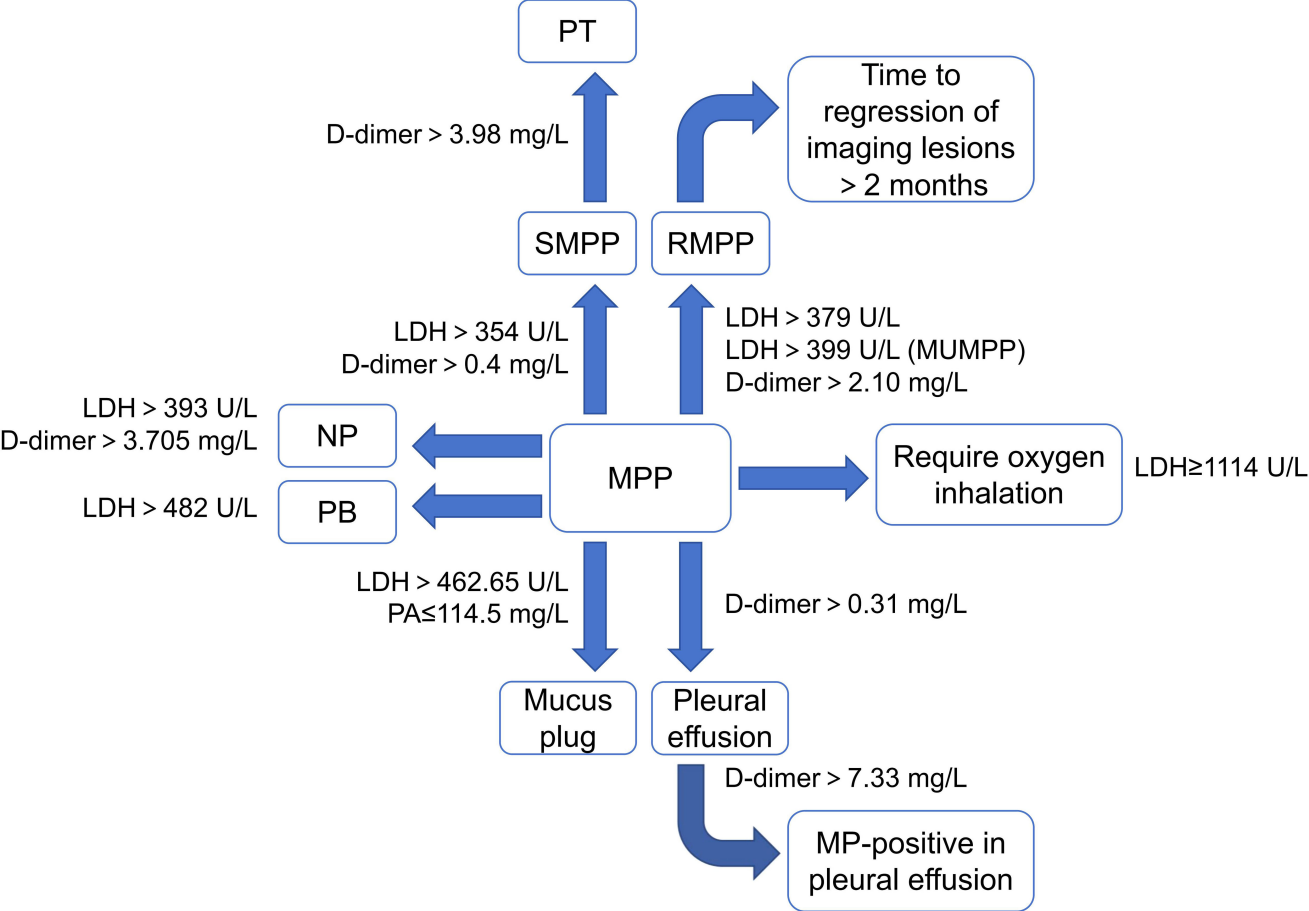
Characteristics of other protein-based markers in MPP and its different prognoses. (MPP, Mycoplasma pneumoniae pneumonia; SMPP, severe MPP; RMPP, refractory MPP; NP, necrotizing pneumonia; PT, pulmonary thrombotic; PB, plasticoid bronchiolitis; LDH, lactic dehydrogenase; PA, prealbumin).

In addition, albumin (Alb) and prealbumin (PA) have also been shown to be associated with the progression of MPP. Both are mainly produced by the liver. In addition to maintaining pH and colloid osmolality, Alb also has anti-inflammatory, antioxidant, and anticoagulant effects. Compared with Alb, the half-life of PA is relatively short and it mainly transports thyroxine and vitamin A. The sensitivity of PA to predict infection is higher than that of Alb (Ranasinghe et al., 2022). Deng et al (Deng et al., 2023). found that the level of Alb levels in children with MPP was negatively correlated with MP-DNA, IL-6, IL-10, TNF-α and INF-γ. PA ≤144.5 mg/L in children with MPP is an independent predictor of mucus plug formation (Zhang et al., 2021) (**Figure 3**).

## 4 Cytokine-based markers

### 4.1 Interleukin

IL is a group of small signal molecules secreted by activated immune cells and some non-immune cells. They are an important part of humoral immunity and cellular immunity. After MP infection, they exert their biological effects by binding to specific receptors on the membrane of target cells and are closely related to the synthesis of biomarkers such as CRP (Linge et al., 2022). Abnormally elevated IL-25 and IL-33 were present in children with MPP, and both were negatively correlated with the children’s lung function-related indices such as tidal volume and time to peak tidal expiratory flow/total expiratory time ratio (Wang et al., 2022). Children with SMPP tend to have lower levels of IL-2 and higher levels of IL-6, IL-8, IL-10, and IL-17 than children with GMPP (Han et al., 2024; Leerach et al., 2024; Wang et al., 2024). The levels of IL-6 and IL-10 in bronchoalveolar lavage fluid (BALF) were also significantly higher in children with a high MP-DNA load than in those with a low MP-DNA load (Deng et al., 2023). In the comparison of macrolide-resistant MPP (MRMPP) and macrolide-sensitive MPP (MSMPP), the levels of IL-13 and IL-33 in the MRMPP group were several times higher than those of MSMPP (Wu et al., 2021). Studies have shown that IL-2, IL-6, and IL-10 are independent risk factors for SMPP (Han et al., 2024; Wang et al., 2024). IL-2 >57 pg/mL and IL-6 >55.835 pg/mL as the diagnostic cut-off values for SMPP had high sensitivity and specificity, with AUC of 0.923 and 0.874, respectively (Han et al., 2024). IL-17α ≥13.4 pg/mL and IL-18 ≥472.0 pg/mL could also independently predicted RMPP, with AUC of 0.81 and 0.85, respectively (Gao et al., 2023). IL-8 >2721.33 pg/mL can independently predict the occurrence of PB in children with MPP (Zhong et al., 2021). In addition, MP infection can cause airway hyperresponsiveness, which carries the risk of developing into bronchial asthma if the treatment is not timely. Such children often have higher levels of IL-6 (Shi et al., 2022).

**Figure 4:**
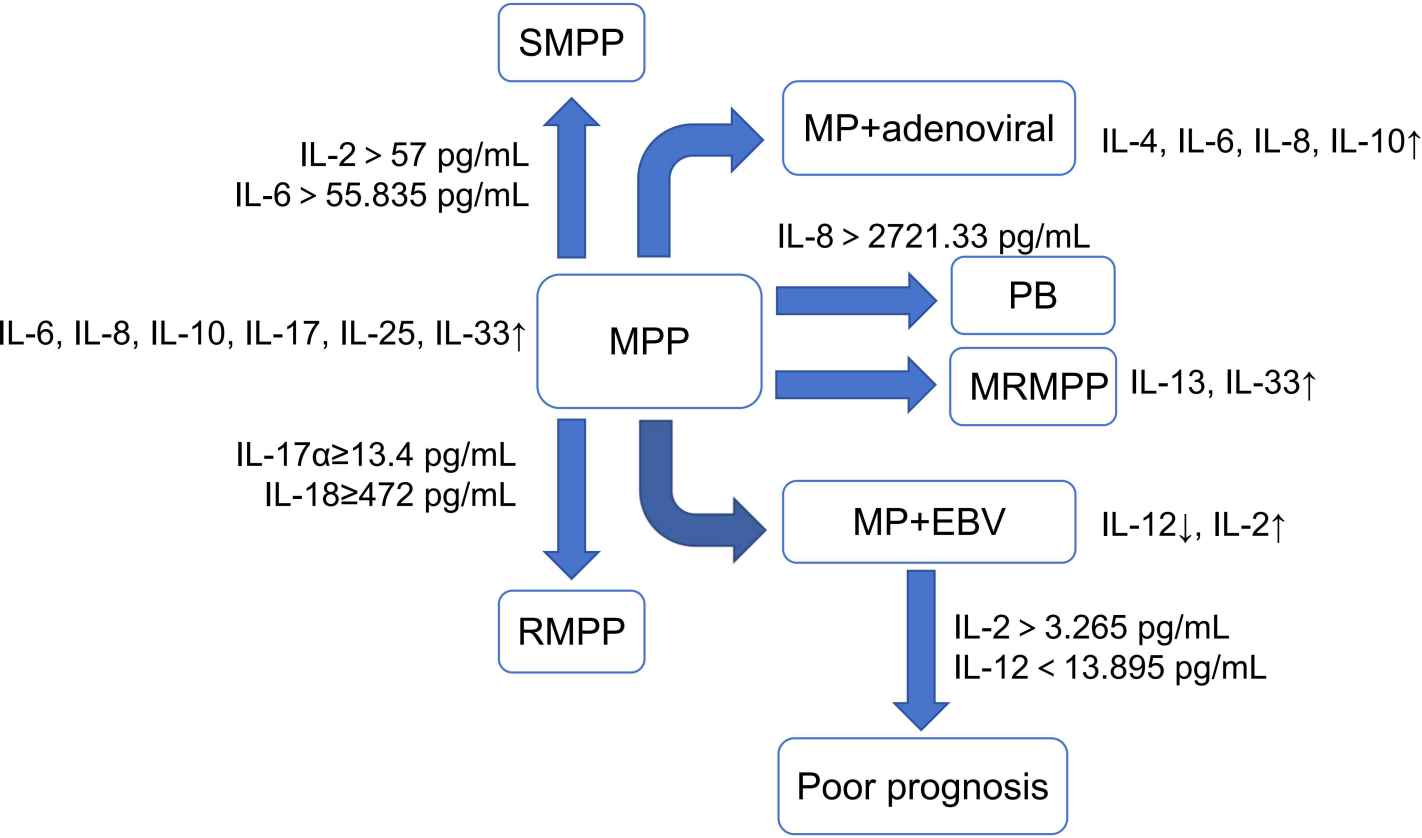
Characteristics of Interleukin in MPP and its different prognoses. (MPP, Mycoplasma pneumoniae pneumonia; SMPP, severe MPP; RMPP, refractory MPP; PB, plasticoid bronchiolitis; MRMPP, macrolide-resistant MPP; EBV, Epstein Barr virus). ↑, increase; ↓, decrease.

MP combined with viral infection is also common in clinical practice. A study showed that IL-5, IL-6, and IL-10 in serum and BALF of children with adenovirus-infected pneumonia are higher than those of children with MPP (Wei et al., 2024). In MPP combined with adenoviral infection, IL-4, IL-6, IL-8, and IL-10 were significantly increased, with more pronounced elevations in severely ill children. The AUC of IL-6, IL-8, IL-10, and IL-17α combined to predict co-infection with adenovirus in children with SMPP was 0.802 (Yi et al., 2024). A significant increase in IL-2 and a significant decrease in IL-12 occur in MP combined with Epstein Barr virus (EBV) infection, while high IL-2 and low IL-12 are independent risk factors for poor prognosis in such children. The AUC for IL-2 >3.265 ng/L and IL-12 <13.895 ng/L combined to predict a poor prognosis for these children was up to 0.915 (Hao, 2024). However, the trend of IL-2 changes in this study seems to be inconsistent with other studies (Han et al., 2024). Therefore, the level characteristics of IL-2 in children with MPP still need further research to be verified (**Figure 4**).

### 4.2 Chemokine

The process of inflammatory response in MPP is closely related to the migration and recruitment of immune cells (such as T-lymphocytes, neutrophils, and eosinophils) to the inflammation site. It is inseparable from chemokines. Chemokines mainly include two categories: receptors and ligands. Chemokine ligands associated with MP infection include eotaxin, Regulate and activate normal T cell expression and secretion factors (RANTES), C-C motif chemokine ligand 17 (CCL17), C-C motif chemokine ligand 2 (CCL2). Among them, eotaxin mainly tends to bring eosinophils closer to the inflammation site and release eosinophil cationic protein. It will not only directly damage the airway epithelial cells and cause airway hyperresponsiveness, but also induces the release of cysteinyl leukotrienes. Then it triggers airway smooth muscle contraction, increases vascular permeability, and ultimately leads to airway remodeling (Maskey et al., 2022). A study showed that the level of eotaxin in children with MPP was abnormally elevated and the lung function-related indicators of children decreased with the increase of eotaxin (Wang et al., 2022). RANTES can bind to receptors on the surface of WBC (such as T lymphocytes, monocytes) to achieve activation and thus regulate immune responses. MP can induce the production and secretion of RANTES on small airways, which is closely related to asthma due to MP infection (Dakhama et al., 2003). And the degree of elevated RANTES is positively correlated with the severity of the disease (Xu et al., 2024). Zhang et al (Zhang et al., 2023). found that RANTES was an independent risk factor for RMPP, and that 3.74 ng/mL as the diagnostic threshold was able to predict the development of RMPP. CCL17 mainly selectively activates Th2 cells. Zhao et al (Zhao et al., 2024). found that the higher the level of CCL17, the more severe the condition of children with MP infection. The sensitivity and specificity of CCL17>53.51ng/L as the diagnostic cut-off value for MP infection were 69.01% and 87.50%, respectively, with an AUC of 0.813. CCL2 is similar to RANTES in that both chemotaxis a variety of white blood cells to recruit to sites of inflammation. A study showed that CCL2 in serum generally does not change significantly in children with MPP, but it was significantly elevated in BALF. And 0.645 ng/mL as the diagnostic threshold for CCL2 in BALF to predict RMPP had an AUC of 0.94 (Zhu et al., 2023).

The main chemokine receptor associated with MP infection is C-X-C chemokine receptor type 2 (CXCR2), which is mainly expressed on the surface of neutrophils and macrophages. It has been demonstrated that the mean fluorescence intensity of CXCR2 in children with MP is significantly higher than that in healthy children. And it is positively correlated with the severity of the disease (Chen et al., 2022). However, no research has yielded a specific diagnostic cut-off value.

### 4.3 Hepatocyte growth factor

Currently, HGF has been confirmed as a protective factor of lung tissue, mainly derived from endothelial cells, alveolar macrophages, fibroblasts, and activated lung neutrophils, with important roles in lung tissue development and lung injury repair (Sang and Qiao, 2024). Previous studies (Liu et al., 2020; Yuan et al., 2021) have shown that HGF is an independent risk factor for SMPP. The sensitivity and specificity of HGF ≥1169.20 pg/mL to predict SMPP were 88.46% and 61.22%, respectively. And through the dynamic monitoring of HGF, it was found that HGF can rapidly decrease to the normal range when the disease was relieved. So it can be used to evaluate the effect of clinical treatment in order to adjust the treatment plan timely. However, there are fewer correlation studies between HGF and MPP. And the sample sizes involved in these two studies are limited. The specific diagnostic cut-off value still needs to be confirmed by further research.

### 4.4 Autotaxin

Autotaxin is a secretory glycoprotein originating from liver tissue with lysophospholipase D activity, which is mainly involved in cell proliferation, cell migration, lipid metabolism, angiogenesis and inflammation (Nikitopoulou et al., 2021). The study showed that the level of autotaxin in serum and BALF of children with RMPP was positively correlated with the degree of inflammation. The level of autotaxin in children with RMPP is significantly higher than that of GMPP, and showed a trend of being significantly higher in the acute phase than in the recovery phase. The sensitivity and specificity of diagnosing RMPP were >80% with 14.25 mg/L and 10.86 mg/L as the diagnostic thresholds of serum autotaxin and BALF autotaxin respectively (Fu et al., 2022).

## 5 Nucleic acid-based markers

### 5.1 microRNA

miRNA is non-coding RNA with a length of 19-25 nucleotides. Studies have confirmed that miRNA is involved in almost all biological processes of cellular activities, including cell proliferation, migration, inflammation, differentiation and apoptosis (Gan et al., 2023). Li et al (Li et al., 2024a). showed that miR-34a was highly expressed in children with MPP, especially in SMPP. And it was a risk factor for poor recovery and its AUCs for recognizing MPP, distinguishing between mild and severe disease, and predicting poor recovery were 0.873, 0.788, and 0.821, respectively. miR-492, miR-223, miR-155, miR-1323, and miR-23a are also significantly upregulated in children with MPP. Among them, the expression levels of miR-223, miR-155, and miR-1323 are positively correlated with the severity of the disease. Overexpression of miR-492 can induce macrophages to secrete IL-6, thus playing a role in the inflammatory response of MPP. Overexpression of miR-23a is closely related to airway hyperresponsiveness caused by MP infection, while miR-1323 is an independent risk factor for RMPP complicated with mixed respiratory virus infection. The AUCs of miR-23a, miR-223, and miR-155 for predicting MP infection are 0.728, 0.772, and 0.850, respectively, with high sensitivity and specificity (Shi et al., 2022; Jia et al., 2023; Ren et al., 2023; Xu et al., 2024; Zhao et al., 2024). This suggests that inhibiting the expression of miR-34a, miR-492, and other miRNAs may serve as a clinical means to alleviate disease progression in severe or refractory cases of MPP. Conversely, serum levels of miR-29c and miR-146a are significantly downregulated in children with MPP. The combined diagnosis of MPP using these two miRNAs yields an AUC of 0.966, with a sensitivity of 94.6% and a specificity of 89.2% (Wang et al., 2023). Furthermore, overexpression of miR-146a in alveolar macrophages has been shown to reduce levels of proinflammatory cytokines. The high level of miR-146a is associated with the suppression of inflammatory cascade reactions after lung infection and improved survival rates (Yoshikawa et al., 2024; Zhao et al., 2024). Additionally, miR-146a can serve as a biomarker for predicting treatment response (Wang et al., 2024).

### 5.2 Genetic polymorphism

Recent researches have found that the genetic polymorphisms of various immune and inflammation-related factors are associated with the susceptibility and prognosis of MP. Orosomucoid 1-like 3 (ORMDL3) is a gene located on chromosome 17, containing 2 introns and 3 exons. It encodes a transmembrane protein located on the endoplasmic reticulum membrane, regulating Ca^2+^ concentration, which leads to the unfolded protein response and subsequently induces inflammatory reactions (Guo et al., 2022). Liu et al (Liu et al., 2024). reported that the GG genotype at the rs4794820 locus and the TT genotype at the rs7216389 locus of the ORMDL3 gene may be potential factors contributing to severe MP infection-induced asthma. The apolipoprotein E (ApoE) gene, located on chromosome 19, encodes a protein consisting of 299 amino acid residues. Besides its involvement in lipid metabolism and cholesterol transport, ApoE is also associated with immune response, antioxidation, and other processes (Khalil et al., 2021). The IL-8 gene, located on chromosome 4, harbors multiple single nucleotide polymorphism loci (Hodeib et al., 2021). According to research conducted by Ni et al (Ni et al., 2023), both the CC genotype and allele C of the ApoE gene at the rs429358 locus, as well as the AA genotype and allele A of the IL-8 gene at the rs4073 locus, can influence the susceptibility to MPP and its prognosis by regulating protein expression.

## 6 Imaging-based markers

MPP often manifests as mild symptoms accompanied by severe lung imaging changes. Therefore, more intuitive lung imaging data also serves as a crucial biomarker for diagnosing MPP and assessing its severity. The length and area of lung parenchymal lesions measured by lung ultrasound can be used to assess the extent of lesions in children with MPP (Guo et al., 2023). Furthermore, there is a correlation between lung imaging findings and serological indicators. According to one study (Wang et al., 2021), children with MPP exhibiting interstitial pneumonia on imaging tend to have more pronounced changes in CRP, while those with lung consolidation show a more significant increase in D-dimer and fibrinogen levels. Huang et al (Huang et al., 2024). analyzed the clinical manifestations and outcomes of MPP children with different imaging features, finding that compared to bronchopneumonia, children with consolidation/atelectasis often exhibit severe abnormalities in clinical manifestations and laboratory indicators. These children also tend to have poorer outcomes, being more prone to developing RMPP, NP, and BO. Additionally, MPP children who have already developed NP may retain imaging manifestations of atelectasis even after treatment (Hou et al., 2025). Besides conventional imaging, CT radiomics has begun to be studied by many scholars. This approach involves extracting a large amount of high-throughput information from medical images through deep analysis, which radiologists often cannot identify or quantify visually. Both Wang et al (Wang et al., 2022). and Li et al (Li et al., 2025). employed methods like SelectKBest and Lasso to screen radiomic features. These radiomic features, when combined, demonstrate high accuracy in distinguishing between bacterial pneumonia and MPP. Moreover, the CT score represents a unique way of presenting CT image results. Lu et al (Lu et al., 2024). determined the CT score based on the degree of ground-glass opacity changes in the lung lobes, assigning specific weight ratios to different CT findings (such as ground-glass opacity, paving stone sign, and consolidation). The results indicate that the CT score has a certain predictive value for RMPP, albeit with limited sensitivity and specificity. Another study utilized AI to quantify lung lesion volumes on CT images, which has been initially proven as a potential biomarker for RMPP (Qian et al., 2024). Among various CT image presentation methods, the CT score is easy to implement, whereas CT radiomics and AI-based CT image quantification are more challenging to promote in clinical settings due to their complex processing workflows.

Furthermore, as previously mentioned, children with MPP, especially infants, often exhibit airway hyperreactivity and varying degrees of lung function alterations. Lung ventilation imaging stands as an effective tool to assess the pulmonary function of these patients. There are three primary methods of lung ventilation imaging: radionuclide lung ventilation imaging, computed tomography lung ventilation imaging, and MRI lung ventilation imaging. Compared to the other two methods, MRI lung ventilation imaging offers advantages such as higher spatial resolution and the absence of radiation harm (Peiffer et al., 2024). In December 2022, the Food and Drug Administration approved the use of hyperpolarized (HP) ^129^Xe gas as the first group of HP MRI contrast agents for lung ventilation imaging. However, the slow production speed, high cost, and incompatibility with low-field MRI scanners of HP ^129^Xe gas have hindered its widespread use, driving the development and validation of a series of proton-HP gases. Currently, validated and feasible options include HP butane gas (Ariyasingha et al., 2024b) and hyperpolarized diethyl ether gas (Ariyasingha et al., 2024a). Although they are not yet widely used in clinical settings, their characteristics of mass producibility, affordability, and compatibility with any MRI scanner suggest that they will facilitate the application of magnetic resonance lung ventilation imaging in MPP in the future.

## 7 Multi-indicator joint predictive models

Although some indicators have demonstrated good applicability, the combined use of multiple indicators in clinical practice can significantly improve diagnostic sensitivity and specificity (**Table 1**), enabling a more accurate understanding of the patient’s condition. Clinical prediction models based on big data and multiple algorithms (such as logistic regression and random forests) further assist medical professionals in making better clinical decisions (**Table 2**).

**Table 1:** Diagnostic efficacy of multi-indicator joint application.

Reference	Case number	Indicators	Diagnosis	AUC	95%CI	Sensitivity	Specificity
(He et al., 2024)	248	SAA, CRP, NLR, PLR	SMPP	0.874	0.801-0.928	82.61%	79.73%
(Wang and Gong, 2024)	320	CD3^+^, CD4^+^, CD8^+^, CD4^+^/CD8^+^, IL-8, IL-10, IL-13, IFN-*y*	MPP	0.924	0.935-0.972	94.76%	90.48%
(Yao et al., 2023)	128	MP-DNA, CD4^+^	RMPP	0.828	0.752-0.904	96.88%	68.75%
(Wang and Wang, 2022)	129	CD3^+^, CD4^+^, CD8^+^	PB	0.862	0.774-0.950	87.50%	71.43%
(Shi et al., 2022)	106	miR-23a, FeNO, IL-6	MPP with airway hyperresponsiveness	0.840	0.771-0.904	86.84%	72.06%
(Yi et al., 2024)	201	CRP, PCT, LDH, Neu%, D-dimer	SMPP	0.977	0.955-0.999	96.00%	93.00%
(Yi et al., 2024)	201	IL-6, IL-8, IL-10, IL-17a	SMPP	0.802	0.719-0.885	89.80%	47.30%
(Hao, 2024)	495	IL-2, IL-12	poor prognosis	0.915	0.858-0.971	85.10%	84.30%
(Yuan et al., 2021)	105	LDH, D-dimer, HGF	SMPP	0.941	0.886-0.997	80.77%	97.96%
(Chen et al., 2022)	118	CD162, CDCR2, CDCR4	SMPP	0.881	0.801-0.961	88.90%	83.30%
(Zhang et al., 2023)	70	IL-6, RANTES	RMPP	0.824	0.688-0.959	79.41%	83.33%
(Zhao et al., 2024)	143	miR-223, miR-155, CCL17	MPP	0.941	0.889-0.974	88.73%	88.89%
(Ren et al., 2023)	100	miR-1323, IL-6	MP+viral infection	0.902	0.826-0.952	86.00%	86.00%
(Wang et al., 2023)	93	miR-29c, miR-146a	MPP	0.966	–	94.60%	89.20%
(Liu et al., 2024)	172	CX3CL1, CD40L, TGF-β1	poor prognosis	0.900	0.812-0.956	80.77%	87.04%

**Table 2:** Predictive models for joint application of multiple indicators.

Reference	Online predictive models web site	Target for projections	Indicators	Case number	training set		validation set	
					AUC	95% Cl	AUC	95% Cl
(Guo et al., 2023)	https://zhxylxy0160128.shinyapps.io/Nomogram/	Virus vs MP	age, fever, PCT, WBC, Lym#, Eos#	792	0.859	0.764-0.954	0.820	0.671-0.969
(Zeng et al., 2024)	–	COVID-19 vs MP	age, gender, ESR, D-dimer	590	0.858	0.827-0.888	0.794	0.729-0.859
(Chen et al., 2024)	https://dxonline.deepwise.com/prediction/index.html?baseUrl=%2Fapi%2F&id=42468&topicName=undefined&from=share&platformType=wisdom	Influenza vs MP	Lym#, Eos, HFC, PLT, PLR, Mon%	423	0.995	–	0.893	–
(Rao et al., 2024)	–	MUMPP	the highest temperature before admission, Neu#, CRP, PCT, pleural effusion	224	0.825	0.755-0.894	0.828	0.729-0.928
(Zhang et al., 2024)	https://ertongyiyuanliexiantu.shinyapps.io/SMPP/	SMPP	age, AGR, NLR, CRP, ESR, MPV, coinfection, pleural effusion, primary disease, fever days ≥ 7, wheeze	526	0.876	0.840-0.913	0.839	0.755-0.924
(Li et al., 2024)	–	SMPP	age, decreased sounds of breathing, SF, LDH, incidence of co-infection, respiratory rate, fever duration, days of hospital-stay	1332	0.862	0.839-0.886	–	–
(Shen et al., 2022)	–	SMPP	CRP, LDH, D-dimer	299	0.881	0.843-0.918	0.777	0.661-0.893
(Shen and Sun, 2024)	–	RMPP	fever duration, pleural effusion, WBC, NEP, CRP, LDH, NLR, SUA	369	0.956	0.937-0.974	–	–
(Liu et al., 2022)	–	RMPP	CRP, LDH, pleural effusion, consolidation size/BSA	90	0.955	0.919-0.978	0.916	0.838-0.964
(Cheng et al., 2020)	–	RMPP	LDH, Alb, Neu%, hyperthermia	219	0.884	0.823-0.945	0.881	0.807-0.955
(Li et al., 2023)	–	RMPP	age, fever duration, Lym#, D-dimer, and radiological imaging change	517	0.907	–	0.964	–
(Pei and Luo, 2024)	–	RMPP	BMI, fever duration, WBC, Neu#, CRP, NLR, PLR	338	0.963	0.946-0.981	–	–
(Luan et al., 2023)	–	airway mucus plug	age, pleural effusion, D-dimer, Plasma IFN-γ	263	0.817	0.747-0.889	–	–
(Liu et al., 2024)	–	BO	days of hospital-stay, fever duration, pulmonary hypotension, Neu%, highest LDH, SF, highest CRP, PaO_2_, PaO_2_/FiO_2_, pleural effusion, et al.	116	0.904	0.874-0.936	0.823	0.776-0.878
(Zhang et al., 2023)	–	PB	cough duration, presence of fever before bronchoscopy, extrapulmonary complications, pleural effusion, LDH	120	0.944	0.779-0.962	–	–
(Zhao et al., 2022)	–	PB	peak body temperature, Neu%, PLT, IL-6, LDH and pulmonary atelectasis	547	0.813	0.769-0.856	0.895	0.847-0.943
(Jia et al., 2024)	https://ertong.shinyapps.io/DynNomapp/	pulmonary consolidation	age, fever duration, Lym#, CRP, SF, CD8^+^ T lym%, CD^4+^ T lym%	491	0.902	0.871-0.933	0.883	0.809-0.956
(Xie et al., 2024)	–	PT	NLR, IL-6, D-dimer, pleural effusion, NP	175	0.912	0.871-0.952	–	–
(Luo and Wang, 2023)	–	NP	MP + bacterial infection, chest pain, LDH, CRP, D-dimer, fever	252	0.870	0.813-0.927	0.843	0.757-0.930
(Wang et al., 2023b)	–	Whether BAL treatment is needed	fever duration, CRP, D-dimer, pleural effusion	202	0.915	0.827-0.938	0.983	0.912-0.996

### 7.1 Early differential diagnosis models for MPP

The study on early differential diagnosis model of MPP primarily focuses on distinguishing it from viral infections. Lin et al (Lin et al., 2025). conducted a retrospective review of 264 children with MPP and 72 children with viral pneumonia. Through least absolute shrinkage and selection operator (Lasso) regression analysis and Logistic regression analysis, they identified four factors: TNF-α/IL-10, age, IL-8, and PCT, to construct a predictive model. After analysis, the predictive model demonstrated a good accuracy in distinguishing between viral pneumonia and MPP, with a C-index of 0.878 and an AUC of 0.875. Guo et al (Guo et al., 2023). primarily used peripheral blood parameters as variables, incorporating five independent predictors: age, fever, PCT, WBC, Lym, and eosinophil (Eos). Both the training and validation sets achieved an AUC greater than 0.8. Zeng et al (Zeng et al., 2024). developed a predictive model based on age, gender, erythrocyte sedimentation rate (ESR), and D-dimer, which also demonstrated excellent performance in terms of discrimination, calibration, and clinical application value. Some scholars have further refined the scope of differentiation by constructing differential diagnosis models between MP and adenovirus, influenza virus, and COVID-19. Among them, the prediction model with age, severe pneumonia, bilateral pneumonia, ground-glass attenuation, consolidation, atelectasis, CRP, and LDH as the main variables achieved an AUC of 0.866 (95%CI: 0.831-0.901) for distinguishing adenovirus from MP (Zhang et al., 2022). The prediction model using lymphocyte count (Lym#), platelet count (Pla#), eosinophil percentage (Eos%), monocyte percentage (Mon%), high fluorescence intensity cells, and PLR as the primary variables yielded an AUC of 0.995 for discriminating between influenza virus and MP (Chen et al., 2024). And after normalization, this model has the best efficacy among all models. Lastly, the prediction model focusing on age, CRP, IL-6, and PCT as key variables resulted in an AUC of 0.80 (95%CI: 0.69-0.91) for differentiating COVID-19 from MP (Zhou et al., 2024). In addition, the clinical definition of MUMPP is that those who show no improvement or even further deterioration in their condition after 72 h of regular treatment with macrolide antibiotics. By assessing such patients upon admission using a predictive model, early identification of MUMPP can be achieved, which is beneficial for improving early treatment efficacy. Rao et al (Rao et al., 2024). developed a MUMPP prediction model based on the highest temperature before admission, pleural effusion, neutrophil count (Neu#), CRP, and PCT, which demonstrated excellent predictive performance.

### 7.2 Predictive models for SMPP

Based on MP-IgM, Eos%, eosinophil count (Eos#), ESR, and PA, Chang et al (Chang et al., 2022). developed a nomograph prediction model with an AUC of 0.777 for predicting SMPP. Zhang et al (Zhang et al., 2024). constructed a prediction model with relatively better predictive performance (AUC=0.867) based on age, albumin to globulin ratio (AGR), NLR, CRP, ESR, mean platelet volume (MPV), comorbid infections, pleural effusion, primary disease, duration of fever, and wheezing. Additionally, they established an online dynamic nomogram to facilitate clinical application. Li et al (Li et al., 2024). built a nomogram prediction model for SMPP based on the age, decreased breath sounds, respiratory rate, duration of fever, hospital stay, incidence of mixed infection, SF, and LDH of 1332 MPP children. The predictive performance of this model was similar to that established by Zhang et al (Zhang et al., 2024).

### 7.3 Predictive models for RMPP

Cheng et al (Cheng et al., 2020). used the LASSO regression model to determine optimal predictors (LDH, Alb, Neu%, hyperthermia) and constructed a nomogram predicting RMPP with an AUC of 0.884 (95%CI: 0.823-0.945). Shen et al (Shen and Sun, 2024). found that days of fever, pleural effusion, and levels of WBC, Neu#, LDH, CRP, NLR, and serum uric acid (SUA) were independent predictors of RMPP. Based on the criteria of fever lasting over 10.5 days, presence of pleural effusion, WBC >10.13×10^9^ cells/L, Neu# >6.43×10^9^ cells/L, CRP >29.45 mg/L, LDH >370.50 U/L, NLR >3.47, and SUA <170.5 μmol/mL, they constructed a prediction model for RMPP. The average AUC of the nomogram was 0.956 (95%CI: 0.937-0.974). Age, duration of fever, Lym#, D-dimer, and lung imaging score were used as variables to construct a predictive nomogram, and the AUC for predicting RMPP was 0.907 (Li et al., 2023). Based on this, Pei et al (Pei and Luo, 2024). incorporated additional indicators such as body mass index (BMI), WBC, Neu#, Pla#, NLR, PLR, CRP, and PCT, which increased the AUC of the model for predicting RMPP to 0.963 (95%CI: 0.946–0.981). Combining clinical practice, literature research, and regression analysis, Shen et al (Shen et al., 2022). constructed a predictive model using CRP, LDH, and D-dimer as predictors, achieving an AUC of 0.881 (95%CI: 0.843–0.918) for predicting RMPP. Liu et al (Liu et al., 2022). based on CRP and LDH, integrated lung ultrasound-related indicators such as pleural effusion and consolidation size/body surface area (BSA), resulting in an AUC of 0.955 (95%CI: 0.919–0.978) for predicting RMPP.

### 7.4 Predictive models for severe comorbidities in MPP

The formation of mucus plugs in the airways of children with MPP is closely associated with RMPP. Luan et al (Luan et al., 2023). developed a nomogram prediction model based on age, pleural effusion, D-dimer, and plasma IFN-*y*, which can accurately predict the formation of bronchial mucus plugs, facilitating timely removal via bronchoscopy. Without timely intervention, it is prone to progress into bronchiolitis obliterans (BO). A regression model constructed with variables such as hospital stay, duration of fever, atelectasis, Neu%, peak LDH, peak CRP, SF, and PaO_2_/FiO_2_, has an AUC of 0.904 for predicting the occurrence of BO, with a sensitivity and specificity of 88% and 83%, respectively (Liu et al., 2024). The accumulation of mucous plugs can also lead to the occurrence of PB. For the prediction of PB, Zhao et al (Zhao et al., 2022). identified 6 variables through Lasso regression, including peak body temperature, Neu%, PLT, IL-6, LDH, and atelectasis, as important predictors for constructing a nomogram. The average AUC of this nomogram was 0.813 (95%CI: 0.769-0.856). Zhang et al (Zhang et al., 2023). achieved a higher predictive performance (AUC=0.944) with a nomogram constructed based on pre-bronchoscopy fever, extrapulmonary complications, pleural effusion, cough duration, and LDH. Children with MPP who are older, have a long duration of fever, decreased Lym#, elevated CRP, elevated SF, increased percentage of CD8^+^ T lymphocytes, and decreased percentage of CD4^+^ T lymphocytes are more likely to develop pulmonary consolidation. A dynamic nomogram model constructed using these variables to predict pulmonary consolidation has an AUC of 0.902 (95%CI: 0.871-0.933) (Jia et al., 2024). Pulmonary necrosis and pulmonary embolism are both serious complications of MPP. Luo et al (Luo and Wang, 2023). constructed a predictive nomogram for pulmonary necrosis using variables such as MP combined with bacterial infection, chest pain, LDH, CRP, duration of fever, and D-dimer. The validation of this model has demonstrated good clinical applicability. Xie et al (Xie et al., 2024). through multivariate logistic regression analysis, found that NLR, IL-6, CRP, LDH, D-dimer, pulmonary necrosis, pleural effusion, and pericardial effusion are all risk factors for embolism in children with RMPP. The nomogram prediction model based on these factors has high accuracy in predicting the risk of embolism.

### 7.5 Predictive models for early bronchoscopic intervention in MPP

“The guideline for the diagnosis and treatment of MPP in children (2023)” stated that bronchial alveolar lavage (BAL) should be performed as early as possible for MPP children suspected of having mucus plug obstruction and PB to reduce the occurrence of complications (National Health Commission of the People’s Republic of China, 2023). Early BAL can help prevent MPP from progressing to SMPP or reduce the severity of SMPP (Wu et al., 2023). Li et al (Li et al., 2024). found that fever duration ≥6.5 days before bronchoscopy, CRP ≥20.94 mg/L, LDH ≥461.5 U/L, and pleural effusion are risk factors for MPP children requiring bronchoscopic intervention. By assigning scores to different factors, when the total score is ≥6 out of 10, the tendency for bronchoscopic intervention is >80%. The higher the score, the greater the likelihood of bronchoscopic intervention. Currently, clinicians often rely solely on imaging to determine whether MPP children should undergo BAL. Atelectasis is a strong indicator for bronchoscopic intervention, but many MPP children with bronchial mucus plugs show lung consolidation on imaging. Wang et al (Wang et al., 2023b). developed a predictive model that assesses the probability of BAL for MPP children with lung consolidation based on fever duration, CRP, D-dimer, and pleural effusion. The external validation AUC is as high as 0.983 (95%CI: 0.912-0.996).

**Figure 5:**
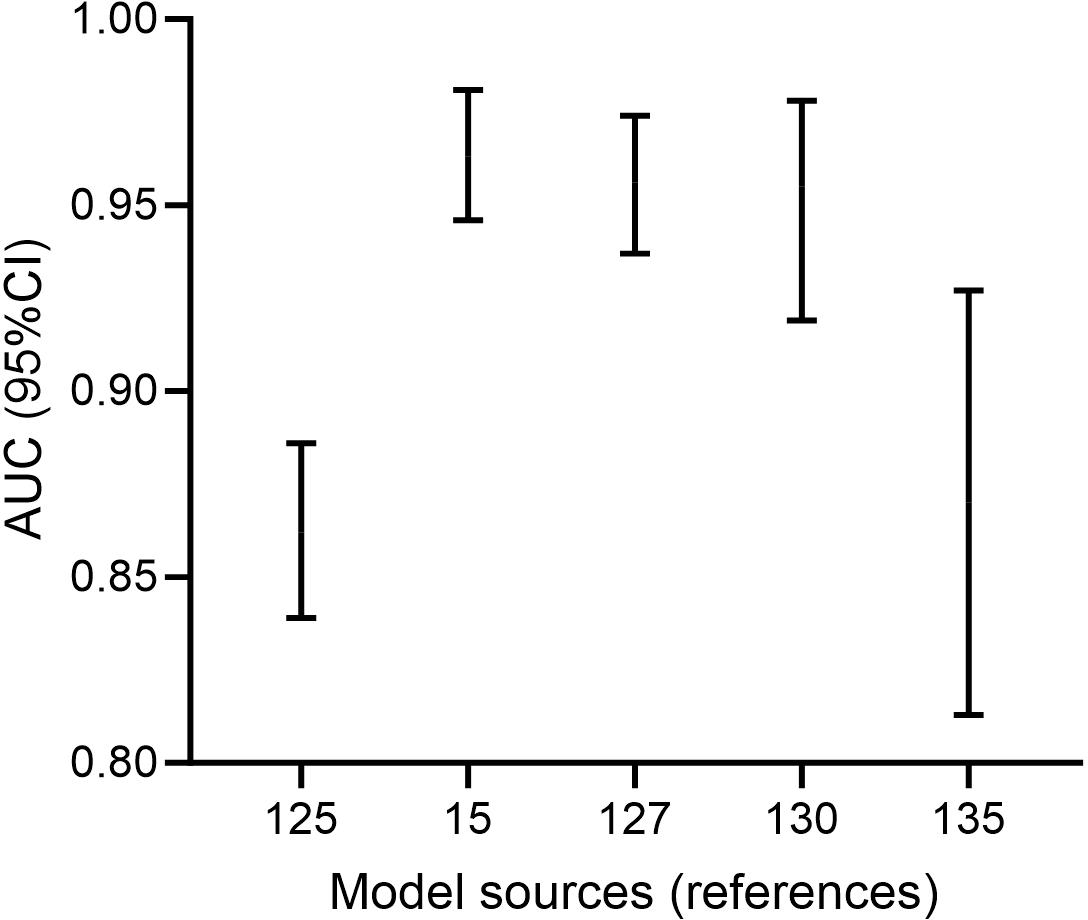
Five predictive models with excellent comprehensive scores. (The two ends of the error bar represent the 95%CI of AUC. The horizontal coordinate decreases from left to right in the rankings of comprehensive scores. As the top-ranked and fifth-ranked models did not provide the corresponding 95%CI for AUC, we included the sixth-ranked and seventh-ranked models in the plot for a supplement.).

To more accurately and objectively compare the effectiveness of different prediction models, we standardized the case numbers across the 20 prediction models included in this study. Specifically, we converted the case numbers for each model into relative proportions (the percentage of each model’s case number relative to the total case number across all models). Since the AUC is already a standardized value, it can be used directly. Considering that clinical prediction models need to balance both sample representativeness and model discrimination ability, we set the weighting ratio to 5:5. We then calculated a comprehensive score for each model (comprehensive score = 50% × AUC + 50% × standardized case number) and ranked them accordingly (**Table 3**). In a study conducted by Shi et al (Shi et al., 2022), they precisely evaluated the effectiveness of a biomarker by comparing the 95%CI of biomarker means between the observation and control groups. Adopting a similar approach, we utilized the 95%CI of the AUC from prediction models to graphically present the top 5 models with higher comprehensive scores (**Figure 5**). Instead of using the variables employed in model construction, we opted for this method because model variables often possess multiple dimensions, such as percentages (e.g., neutrophil percentage), hundreds (e.g., platelet count), and qualitative indicators (e.g., the presence of pleural effusion). These variables might not be suitable for simultaneous representation on a single chart, as demonstrated by Shi et al (Shi et al., 2022). However, by using the AUC and its 95%CI, we can also more accurately compare the AUC levels of different prediction models that aggregate numerous biomarkers, rather than focusing on individual biomarkers.

**Table 3:** Ranking of the effectiveness of different prediction models.

Reference	Target for projections	case number	AUC	Standardized case number	Comprehensive score (weighting 5:5)	ranking
(Chen et al., 2024)	Influenza vs MP	423	0.995	5.36%	0.5243	1
(Li et al., 2024)	SMPP	1332	0.862	16.89%	0.5155	2
(Pei and Luo, 2024)	RMPP	338	0.963	4.29%	0.5029	3
(Shen and Sun, 2024)	RMPP	369	0.956	4.68%	0.5014	4
(Li et al., 2023)	RMPP	517	0.907	6.56%	0.4863	5
(Liu et al., 2022)	RMPP	90	0.955	1.14%	0.4832	6
(Luo and Wang, 2023)	pulmonary consolidation	491	0.902	6.23%	0.4821	7
(Guo et al., 2023)	Virus vs MP	792	0.859	10.04%	0.4797	8
(Zhang et al., 2023)	PB	120	0.944	1.52%	0.4796	9
(Zhang et al., 2024)	SMPP	526	0.876	6.67%	0.4714	10
(Wang et al., 2023b)	Whether BAL treatment is needed	202	0.915	2.56%	0.4703	11
(Xie et al., 2024)	PT	175	0.912	2.22%	0.4671	12
(Zeng et al., 2024)	COVID-19 vs MP	590	0.858	7.48%	0.4664	13
(Shen et al., 2022)	SMPP	299	0.881	3.79%	0.4595	14
(Liu et al., 2024)	BO	116	0.904	1.47%	0.4594	15
(Cheng et al., 2020)	RMPP	219	0.884	2.78%	0.4559	16
(Luo and Wang, 2023)	NP	252	0.870	3.20%	0.4510	17
(Zhao et al., 2022)	PB	547	0.813	6.94%	0.4412	18
(Rao et al., 2024)	MUMPP	224	0.825	2.84%	0.4267	19
(Luan et al., 2023)	airway mucus plug	263	0.817	3.34%	0.4252	20

Nevertheless, we have also identified some shortcomings. Firstly, most current research on MPP-related biomarkers is limited to single-center retrospective studies with a narrow sample scope and limited sample size. Some studies only include a few tens of cases, leading to diagnostic cut-off values that may not have good universality. Secondly, after comparing the effectiveness of these prediction models by assigning appropriate weights to the number of cases and AUC, and displaying the results graphically, we found that the AUC level of the second-ranked model is significantly lower than that of the third, fourth, and sixth-ranked models. Additionally, despite some models having high AUC values, their effectiveness remains relatively low due to inadequate sample sizes (poor sample representativeness). Finally, the established prediction models primarily incorporate variables such as peripheral blood parameters and clinical symptoms/signs, covering a relatively limited range of variable types. Furthermore, some prediction models have not undergone a comprehensive internal development and external validation process (Luan et al., 2023; Zhang et al., 2023; Li et al., 2024; Pei and Luo, 2024; Shen and Sun, 2024; Xie et al., 2024), which may impact the reliability and generalizability of the models to some extent. Future research should aim to enrich prediction models by incorporating additional variables strongly associated with the predicted outcomes, while also including a larger number of cases to enhance sample representativeness. Adequate validation is essential to ensure the usability of the models. By adopting an online prediction model approach, the utilization process of the models will not be complicated due to the appropriate increase in variables, rather than relying solely on a static nomogram format. Finally, the use of new technologies (such as magnetic resonance pulmonary ventilation imaging) may also be able to help the clinical management of MPP. Given the complex clinical detection processes associated with gene-related biomarkers, there is potential to design targeted therapeutic drugs based on the functions of these genes, which may find promising applications in severe and refractory pediatric cases.

## 8 Conclusions

In summary, these involved indicators all have a certain correlation with the severity of MPP. Among them, CRP, PCT, and SAA, which are the most common indicators in clinical practice, exhibit high sensitivity in the early differential diagnosis of MPP. Meanwhile, CRP and LDH can, to some extent, predict the treatment measures that should be taken for children as well as their response to treatment. Affordable and convenient blood routine, and cytokine-based markers detected using enzyme-linked immunosorbent assay, can be used alongside other aforementioned indicators to jointly assess the condition. For detecting complex and expensive biomarkers (such as nucleic acid-based markers), they can be utilized in areas like new drug development for MPP to maximize their application value. However, as we all know, it is difficult to accurately achieve the purposes of diagnosis, prediction, and evaluation by relying on a single indicator alone. The combined detection of multiple indicators can greatly improve the sensitivity and specificity of diagnosis, maximizing its clinical application value. Some studies have established joint prediction models based on multiple indicators, and some scholars have even designed an online prediction model, vastly improving the application efficiency of these models and greatly assisting pediatricians.
